# Wettability of polytetrafluoroethylene surfaces by plasma etching modifications

**DOI:** 10.1371/journal.pone.0282352

**Published:** 2023-03-31

**Authors:** Hyomin Kang, Sang Hyuk Lee, Kiwoong Kim

**Affiliations:** 1 Department of Mechanical Engineering, Hannam University, Daejeon, Republic of Korea; 2 Department of Nuclear Equipment Qualification & Safety, Korea Institute of Machinery & Materials, Daejeon, Republic of Korea; University of Pittsburgh, UNITED STATES

## Abstract

Superhydrophobic surfaces (SHS) are attracting attention in many fields owing to their excellent advantages such as anti-freezing, corrosion prevention, and self-cleaning. However, to modify the surface structure, environmental pollution caused by complex processes and chemical treatment must be considered. In this study, the surface of polytetrafluoroethylene (PTFE) was plasma-treated using oxygen and argon plasma to change the surface structure without a complicated process. The PTFE surface was treated in two ways: plasma etching (PE) and reactive ion etching (RIE). The contact angle of the conventional PTFE surface was 113.8 ± 1.4°, but the contact angle of the manufactured surface was 152.3 ± 1.7° and 172.5 ± 1.2°. The chemical composition and physical structure of the samples produced were compared. The treated specimens had the same chemical composition as the specimen before treatment and exhibited differences in their surface structures. Therefore, it was determined that the change in the water repellency was due to the surface structure. After PE treatment, the specimen surface had a mountain range-like structure, and the RIE specimen had a more detailed structure than the PE specimen. The contact rate of water droplets decreased due to the difference in the structure of the specimen before and after treatment, and the increase in the surface contact angle was manifested. In order to confirm that the plasma treatment reduces surface energy, the shape of the liquid collision was observed using a high-speed camera, and the contact time was calculated to confirm water repellency. The contact time of the PE and RIE specimen was 24 milli-second (ms) and 18 ms, respectively. The high contact angle and low sliding angle of the RIE specimen made it easy to restore surface cleanliness in a self-cleaning experiment using graphite.

## Introduction

The low wettability shown on some solid surfaces is an important natural phenomenon that has been studied for a long time because it offers excellent anti-freezing, self-cleaning, and anti-corrosion properties [1–9]. These characteristics, which can be observed in animals and plants such as lotus leaves and water striders [10–13], are expressed due to the nano/micro-sized structures on the surface and chemical coatings with reduced surface energy [2, 14–16]. Due to the nano/micro structures and chemical coatings of the surface, the surface has low surface energy. This surface is called a superhydrophobic surface, and it has a contact angle of 150° or more and a sliding angle of 10° or less. SHS is applied to solar cells, vehicle glass, and fibers because micropollutants can be removed using only water droplets [17, 18].

With increasing efforts devoted to realize SHSs, researchers have suggested various methods, including plasma treatment, particle deposition, sol–gel method, vapor deposition, and casting methods [17, 19–22]. Among them, plasma treatment is one of the methods being studied in various fields [23–25]. Plasma treatment is a method of transforming into a plasma state by applying a strong electric field to gases (O_2_, CHF_3_, SH_6_, Ar) and etching the surface using ions [26, 27]. Given that plasma treatment using oxygen and argon does not require chemical post-treatment, environmental pollution does not occur and the etching process becomes very simple. In addition, plasma treatment has the characteristic that the surface can be changed to hydrophilic or hydrophobic by changing the surface structure [28, 29]. In this study, SHS is intended to be produced using the plasma.

Among various types of plasma treatment methods, plasma etching (PE) and reactive ion etching (RIE) are the most representative methods in surface etching. These two types of plasma processing depend on which of the upper or lower substrates present in the plasma chamber is applied with power. In this case, the PE method applies power to the upper substrate, and the influence of ions is smaller than that of the RIE method. Conversely, in the RIE method, power is supplied to the lower substrate of the chamber, and self-bias is formed on the substrate side to increase sheath energy [30]. Given this difference, the surface was treated differently. This study attempted to compare directly the difference in wettability between the PE and the RIE methods.

**Fig 1:**
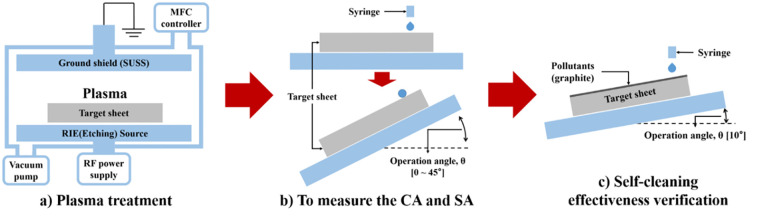
Schematic diagram of surface fabrication and experimental processes. (a) Plasma treatment of PTFE by controlling variables. (b) Measure the contact angle and sliding angle of the surface using a surface tension measuring equipment. (c) Check the self-cleaning effect of the specimen using graphite and water droplets.

Polytetrafluoroethylene (PTFE) is one of the fluororesins composed of carbon and fluorine, and it has excellent heat resistance, chemical resistance, low-temperature resistance, and electrical insulation [31, 32]. In addition, it has a high working temperature and a low coefficient of friction. Hence, it is widely used in aerospace, chemistry, machinery, and architecture. In particular, the low wettability of PTFE can confirm that this object can easily change to SHSs [5, 21, 33–35]. Zhang et al. used Teflon tapes to fabricate SHSs [34]. Zhan et al. used PTFE to create SHSs with a high contact angle [5]. Hossain et al. studied a method to impart functional coatings to heat-sensitive materials such as fabric and paper through atomospheric-pressure plasma polymerization of hexamethyldisiloxane [36]. However, considering that the use of these substances could adversely affect the environment, the surface was treated using oxygen and argon in this study. Saffar et al. reduced surface energy through two processes to impart superhydrophobic properties to the copper surface [9]. However, surface manufacturing using ZnO can have dangerous effects on the environment and living things. In this study, the SHS was produced by treating the specimen as shown in Fig 1(a), and the characteristics of SHS as shown in Fig 1(b) and 1(c) were confirmed.

## Materials and methods

### Materials

A polytetrafluoroethylene (PTFE) specimen (Hyunwoo TC, Korea) was selected as a material via plasma treatment. The selected PTFE specimen has a low surface free energy (20 mNm^-1^ at 20°C) and excellent water repellency before plasma treatment. Accordingly, superhydrophobic feature can be easily imparted to the selected surface. PTFE can be used for various purposes because of its chemical properties (chemical resistance, insulation, etc.). The PTFE specimen has a diameter of 35 mm and a hole diameter of 5 mm inside. The thickness of the specimen is 2 mm. PTFE was washed with a mixed solution of ethanol and de-ionized water at a ratio of 7:3 and dried naturally in a fume hood.

### Surface modification via plasma treatment

In this study, PTFE specimens were separated in two ways. One is through the PE method and the other is through the RIE method. After the washed PTFE specimen is disposed in the vacuum chamber, Ar and O_2_ gas were injected using a mass flow controller (MFC). The flow rate can be adjusted up to 100 sccm via the MFC. The internal pressure is maintained at 300 mTorr, and the surface treatment output is 13.56 MHz. The maximum available RF power for the equipment is 600 W. The diameter of the electrode in the plasma equipment is 150mm, the distance between the electrodes during processing is 50 mm, and the pressure inside the chamber is lowered to 5 mTorr before processing to improve processing accuracy. Oxygen and argon having a purity of 99.99% were used as the gas injected into the chamber.

In the PE method, power is supplied to the upper substrate in the chamber. Conversely, in the RIE method, power is supplied to the substrate under the chamber. In this case, self-bias is formed on the electrode side and the sheath energy increases. Considering that the lower substrate of the chamber is in direct contact with the specimen, the specimen treated in the RIE method, which is directly connected to the lower substrate, is exposed to higher ion energy. The PE method is a method of etching using radical generated in the plasma generation process and may cause a chemical reaction. In this case, isotropic etching occurs and electrical damage is not applied to the specimen. It has the advantage of having high selectivity and etch rate. RIE method is a method that uses a mixture of a chemical method and a physical method. With a method of mixing anisotropy, which is an advantage of physical etch, and high selectivity and etch rate, which are advantage of chemical etch, characteristics can be selectively selected depending on the weight of physical and chemical. As a result, the surface may be treated differently. This plasma treatment method is cleaner and safer than wet etching method. In particular, in this research, the gases used to form plasma in the PE and RIE methods are Ar and O_2_, which are more economical and eco-friendly than other gases (CHF_3_, SH_6_) for manufacturing SHSs.

### Analysis of the physical and chemical characteristics of the fabricated SHP surface

In order to confirm the wettability of the superhydrophobic PTFE, a surface tension measuring equipment (SmartDrop, Femtobiomed, Korea) was used. Water droplets were generated with de-ionized water to measure the surface contact angle and sliding angle. The size of the water droplets for measurement was 10 μL.

To confirm the structural characteristics of the treated sheet, FE-SEM (Sirion MSE10, FEI, Netherlands) was used to analyze the PTFE surface. Given that the PTFE sheet has non-conductive properties, platinum coating was deposited for 30 seconds to prevent charging effect.

SPM (XE-100, Park System, Korea) was used to analyze the roughness of the surface. It was intended to confirm the change in surface shape by comparing the pristine specimens with the treated specimens.

Changes in the chemical composition of PTFE surfaces through plasma treatment were confirmed using an FT-IR spectrometer (IFS66v/s, Bruker Optiks, Germany). The chemical composition of the sheet before treatment, after PE treatment, and after RIE treatment was analyzed.

X-ray Photoelectron Spectrometer (XPS, K-Alpha+, ThermoFisher, USA) analysis of the specimen before and after treatment was conducted to analyze the chemical composition of the surface.

In order to verify the self-cleaning effect of PTFE surface, graphite was used to contaminate the surface and graphite was removed from the surface using only water droplets. Specifically, graphite (≤ 45 μm) was used to contaminate the PTFE surface. The sheets stained with graphite were tilted 5°, and water droplets were dropped on the surface. After that, only water droplets were used to remove the graphite from the surface.

The surface was observed using a high-speed camera built into the surface tension measuring equipment to observe the water droplet impact phenomenon on the surface. The droplets were photographed at intervals of about 1.2 ms, and the movement of the droplets after the collision was compared. The droplets were dropped on the surface using a syringe pump. In this case, the needle from which the droplet fell was placed at a height of 20 mm from the sheet, and the droplet size was 11 ± 0.2 μL.

## Results and discussion

### SHSs treated with PE and RIE methods

Table 1 shows the design of processing parameters for plasma treatment processing. Important parameters in the process are the strength of the power source, process time, type of activator, and flow rate [21, 37]. Each parameter was divided into three levels, and an experiment was designed on the basis of the orthogonal arrangement method. In this case, when the used power is high and the processing time is long, the etching rate of the surface increases, but the temperature in the chamber increases. Therefore, RF power and exposure time were limited to prevent possible damage to the specimen. Ar and O_2_ were selected as gases for plasma generation. Ar and O_2_ are more economical and eco-friendly than other gases (CHF_3_, SH_6_) used to fabricate SHSs. In particular, O_2_ has good reactivity, so it may have greater influence on the etching rate than Ar. Therefore, the flow rate of O_2_ was selected as a parameter, and the flow rate of total gas was separately treated as a parameter. As a result, a total of four parameters were selected, and we tried to identify the most influential parameter in expressing water repellency among these parameters.

**Table 1:** Design of surface treatment parameters for plasma treatment.

Specimen No.	RF Power [W]	Exposure Time [s]	O_2_ gas flow [sccm]	Total gas flow [sccm]
**1**	125	450	30	60
**2**	125	600	40	80
**3**	125	750	50	100
**4**	150	450	40	100
**5**	150	600	50	60
**6**	150	750	30	80
**7**	175	450	50	80
**8**	175	600	30	100
**9**	175	750	40	60

**Fig 2:**
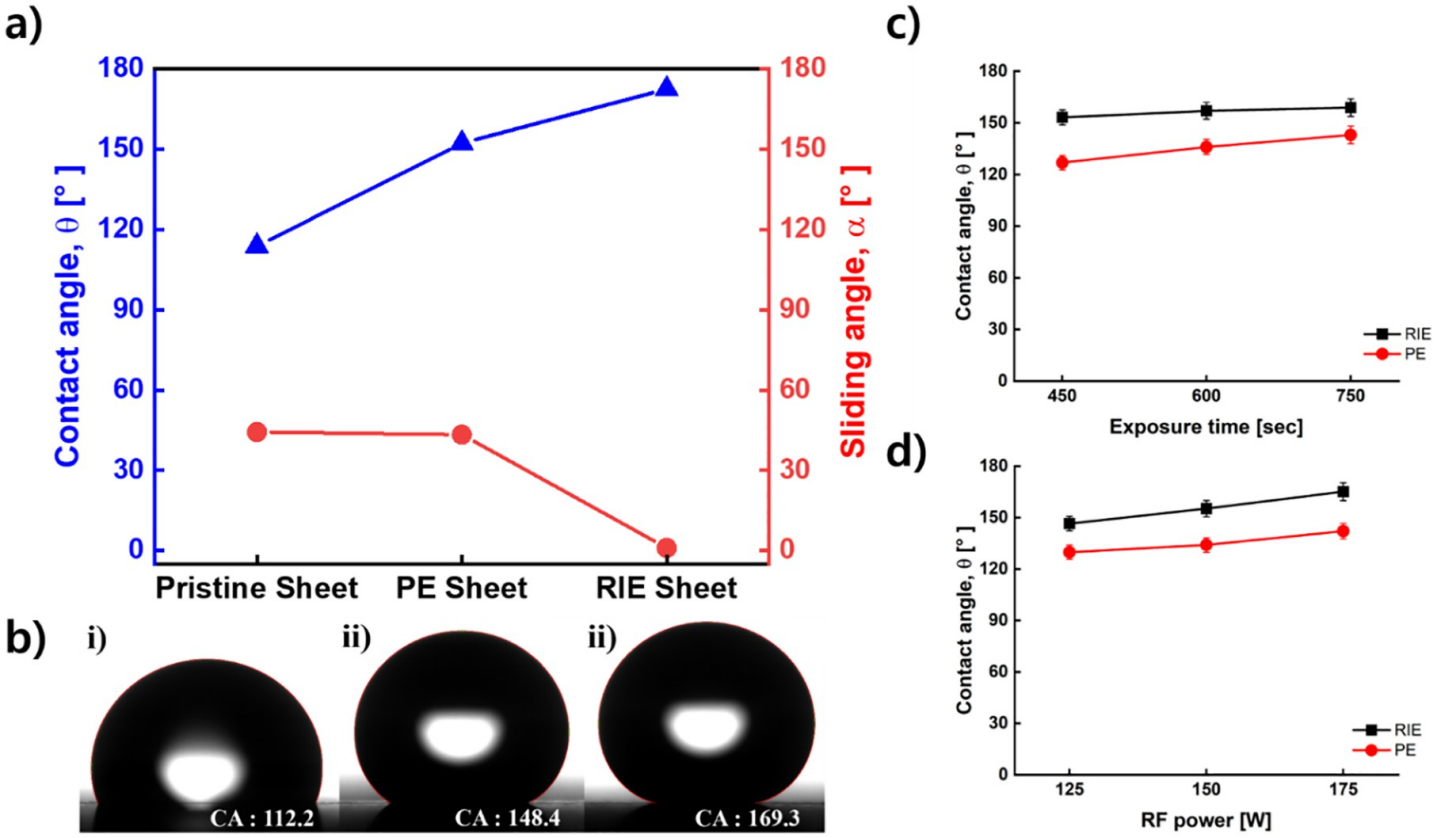
Graph of contact angle and change of contact angle according to slide angle and surface treatment parameters of treated specimen. (a) contact angle and sliding angle graph of before and after treatment (b) i) Before treatment, ii) After PE treatment, iii) After RIE treatment, the image where the droplet is located and the contact angle. (c, d) The contact angle graph according to the surface treatment parameter (RF power, exposure time). The PE-treated specimen is expressed in red and the RIE-treated specimen is expressed in black.

Fig 2(a) summarizes the changes in contact angle and sliding angle of the treated specimen. The maximum contact angle of the PE-treated specimens (PE specimens) was 152.3±1.7◦, and the sliding angle was 43.4±1.4◦. The contact angle of the PE sample was significantly increased compared to that of the untreated specimen (Pristine specimen, 113.8±1.4◦), but the slide angle (44.3±1.4◦) was not significantly changed. Fig 2(c) shows the graph of the contact angle according to the exposure time, which is the most influential parameter in the PE method. The second most influential parameter was RF power, and the influence of exposure time was about 1.7 times that of this parameter. PE and pristine specimens did not form on the surface when water droplets were dropped on the surface, which was caused by the low sliding angle of the two specimens.

Based on the variance of the contact angle according to the change in each variable, an influence comparison through the orthogonal array method was presented. When the RF power level changed, the variance of the contact angle was confirmed by the slope of the graph in Fig 2(c). The slope at this time was 9.3, which was confirmed to be the steepest among the variables. Conversely, it was confirmed that the variance of the contact angle for the total gas flow rate was the smallest at 2.3. As a result, RF power could be selected as the most influential parameter to fabricate high contact angle surface. The most influential parameter in the RIE-treated specimens (RIE specimens) was RF power. Fig 2(c) summarizes the change in contact angle according to RF power. The maximum contact angle of the specimen treated with the RIE method was 172.5±1.2°, and the sliding angle was less than 1°. This low sliding angle prevented water droplets from forming on the surface. In addition, water droplets easily fell at an angle change of 0.5° or less. The optimized specimens in PE and RIE specimens were processed when RF power was 175 W and exposure time was 750 s. In this case, the O_2_ gas flow was 40 sccm and the total gas flow was 60 sccm.

Fig 2(b) shows that water droplets are formed on the sample surface before and after treatment. In this case, pristine and PE specimens maintain a Wenzel state. On the other hand, the RIE specimen changed to the Cassie-Baxter state. This difference in state can be confirmed through changes in the surface sliding angle [38]. The PE specimen showed a slip angle difference of about 1° from the existing specimen, and the RIE specimen showed an angle difference of about 40°. This difference in sliding angles means that the surface structure has changed from the Wenzel state, where droplets are located between the surface structures, to the Cache-Baxter state, which is located on the surface structures. Thus, the surface structure of the RIE specimen can be modeled on the Cassie-Baxter state using the following equation, which can calculate the ratio of water droplets on the surface in contact with the actual surface structure [39].

$$cos\theta_{c}=f_{1}cos\theta -f_{2}$$ (1)

$$f_{1}+f_{2}=1$$ (2)

*θ*_*c*_ in Eq 1 represents the contact angle of the processed specimen, and *θ* represents the contact angle of the pristine specimen. *f*_1_ is the contact rate between the droplet and the surface air layer, and *f*_2_ is the contact rate between the droplet and the surface. According to Eq 2, *f*_1,*PE*_ and *f*_1,*RIE*_ were calculated to be 19.2% and 1.4%, respectively. This result indicates that the surface of contact with the water droplets is small and most of them are in contact with the air layer between the surface structures.

### Surface properties of the treated sheet

**Fig 3:**
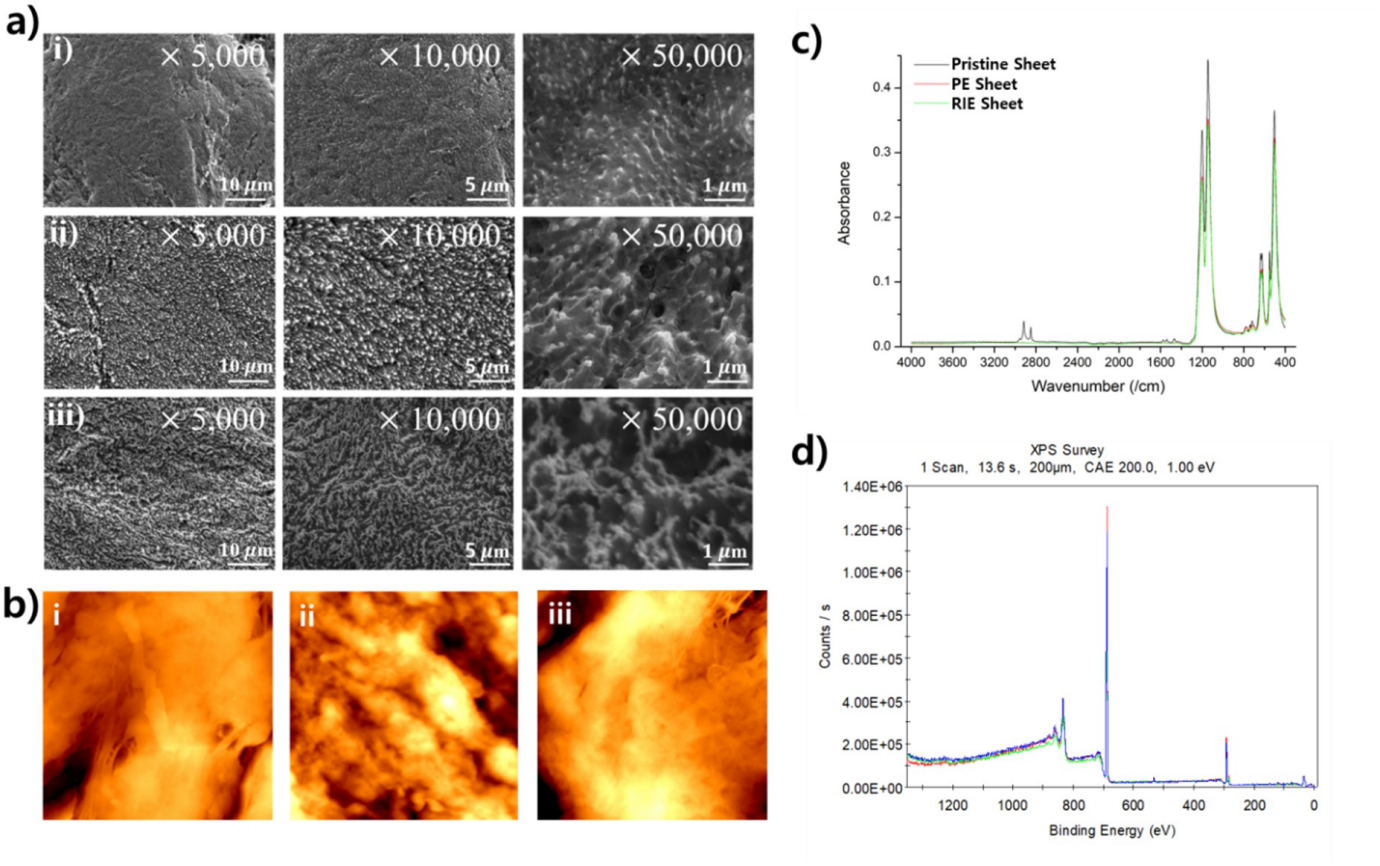
Analysis of surface characteristics. (a) FE-SEM image i) Before treatment, ii) After RIE treatment, iii) After PE treatment. More detailed structures of columnar shapes can be observed on the RIE surface, and leaf vein-like structures can be observed on the PE surface. (b) SPM images of surfaces i) pristine, ii) after PE treatment and iii) after RIE treatment. (c) FT-IR spectrum analysis confirmed no significant difference in the chemical composition of the surface before and after treatment. (d) XPS results of pristine, PE, and RIE Specimens.

The water repellency of a surface depends on its structure and chemical composition. Therefore, the cause of the increase in water repellency of the specimen treated in this study can be found in the change in structure and chemical composition. Fig 3(a) shows FE-SEM images of pristine, PE, and RIE specimens. On the one hand, in Fig 3(b), the PE specimen has a microstructure with the same shape as a leaf vein. On the other hand, the RIE specimen was densely shaped like a small column, as shown in Fig 3(a). In addition, the tip of the column has a spherical shape. The RIE specimen had a finer shape than the PE specimen, and an air layer was formed between these structures. This layer reduced the contact area between the water droplets and the surface, and increased the contact angle and the sliding angle.

The results confirmed that the structural change of the specimen through plasma treatment was formed differently in PE and RIE. This change in surface structure is due to the difference between the PE and RIE methods, which are distinguished by the difference in the position of the substrate with power supplied within the chamber. In this case, the chamber lower substrate is in direct contact with the specimen. As a result, the specimen is exposed to higher ion energy in the RIE method compared to the PE method. The difference in ion energy seems to be the reason why the surfaces are treated differently in the PE and RIE methods. The high ion energy would have caused more etching on the surface and greatly increased the roughness of the surface. Considering that such roughness has a great influence on surface water repellency, the contact angle and the sliding angle have resulted in a large change.

Fig 3(c) shows an SPM image before and after processing. Small cracks were generated on the surface through plasma treatment. This structural change may be clearly seen from the difference between Fig 3(a)i and 3(a)iii, and the generated RIE specimen may have a smaller structure than the specimen before treatment.

PTFE has a contact angle of about 120° before treatment [40, 41], because PTFE is one of the fluororesins containing fluorine. Fluorine coating is one of the efficient methods for imparting water repellency to surfaces. The plasma treatment used in this study physically etched the surface without changing this chemical composition. Therefore, the surface was analyzed using an FT-IR spectrometer to confirm the chemical composition of the surface. Fig 3(c) confirms that no difference was observed in the surface chemical composition before and after the plasma treatment. The bandwidths of 501 cm^-1^, 554 cm^-1^, and 638 cm^-1^ correspond to the shaking, bending, and shaking of CF_2_. And the constant absorption bands of 1145 cm^-1^, 1199 cm^-1^ are found to be due to CF_2_ symmetric stretch vibration modes. Therefore, the contact angle and the sliding angle of the surface changed due to the structural change of the surface.

The XPS anlysis was performed to confirm the change in the chemical composition of the specimen. According to the analysis results of Fig 3(d), it was confirmed that the specimen before treatment had a ratio of C1s:F1s:O1s = 34.5:64.5:1. In addition, the RIE specimen had a ratio of C1s:F1s:O1s = 33.8:64.7:1.5, and the PE specimen had a ratio of C1s:F1s:O1s = 30.4:68.2:1.4. As a result, it was confirmed that the composition of the surface did not show much change after treatment. Therefore, as in FT-IR analysis, the chemical composition of the surface did not significantly affect the water repellency of the surface, and it was confirmed that the structural change of the surface had a higher effect on the water repellency of the surface.

### Water repellency of surfaces treated by using PE and RIE methods

**Fig 4:**
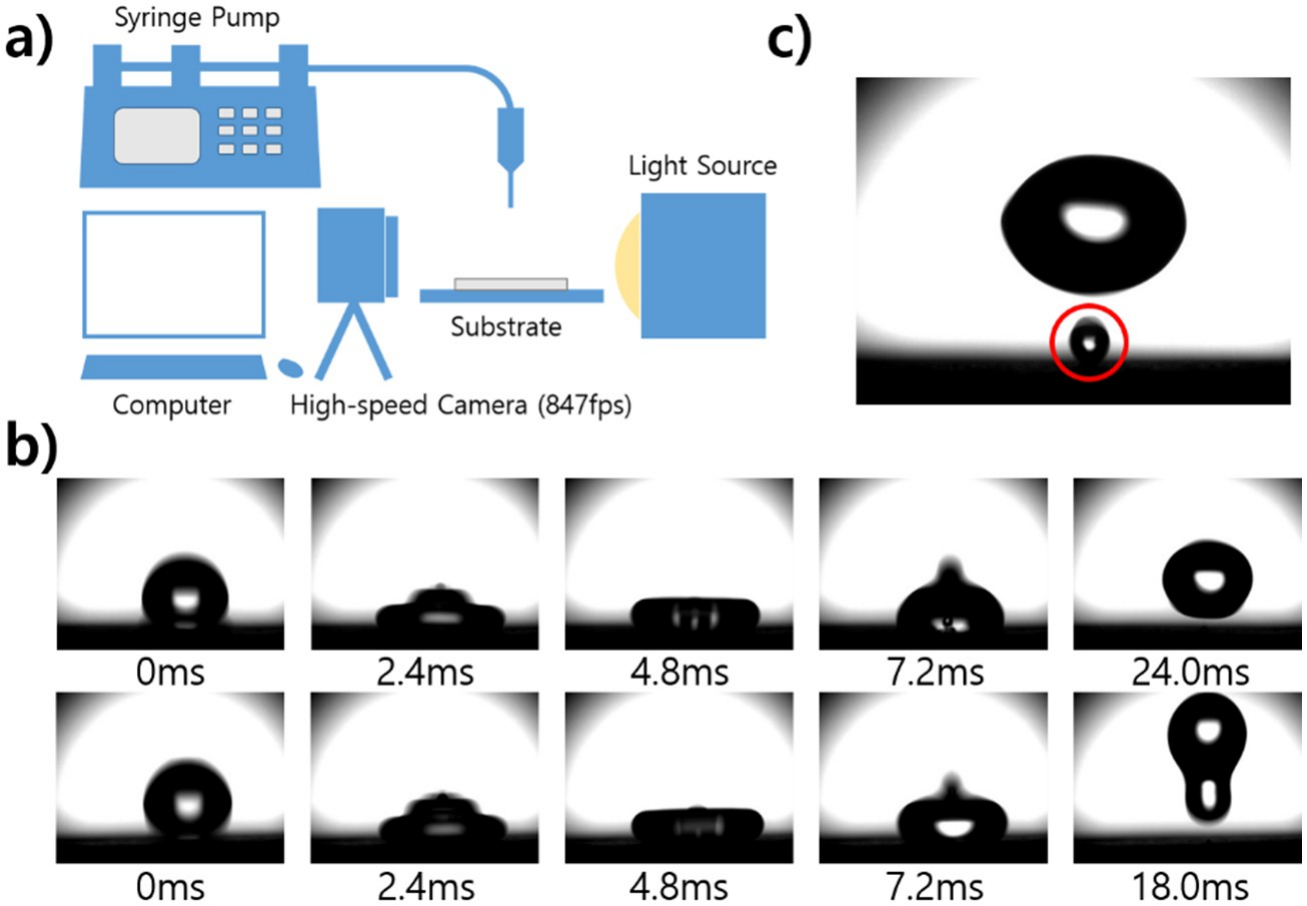
Observation and analysis of droplet collisions. (a) Composition of experimental equipment for droplet collision observation. The needle dropped droplets at a height of 20 mm from the surface and the droplet size is 2.8±0.2 mm. The velocity of the droplet was 1.02 m/s. (b) Results of high speed cameras from contact with droplets to removal from surfaces. Compared with the specimen treated with the PE method, water droplets splashed faster on the specimen treated with RIE. (c) When water droplets bounced from the specimen treated with the PE method, small water droplets are scattered. The small droplets were between 0.4 and 0.5 mm in size.


SHSs have low surface energy, which makes it difficult for liquids to form on the surface. To confirm this property, the equipment was arranged as shown in Fig 4(a). The surface energy of the treated specimen was calculated, and water repellency was confirmed. The water droplet velocity (1.02 m/s), size (2.8 ± 0.2 mm), and density (998.2 kg/m^3^) were controlled. And the energy of the surface was calculated by Eq 3 [42].

$$cos\theta =-1+2\sqrt{\frac{\gamma_{sv}}{\gamma_{lv}}}e^{-\beta {\left(\gamma_{lv}-\gamma_{sv}\right)}^{2}}$$ (3)

*θ* is the contact angle, and *γ*_*sv*_ and *γ*_*lv*_ are the interfacial tension of the solid–gas and the liquid–gas, respectively. *β* was calculated as 0.0001247 (*m*^2^/*mJ*)^2^ in consideration of the used liquid. In this case, the surface tension of the liquid was 75.2 mN/m on average. The surface energy of the specimen calculated through equations was 16.39 mN/m in the specimen before treatment, 0.76 mN/m in the specimen after PE treatment, and 0.02 mN/m in the specimen after RIE treatment. When water droplets collide with a surface, the behavior of water droplets changes with the *We*. *We* is the ratio of kinetic energy to the surface tension of the droplet. Antonini et al. confirmed the behavior of the water droplets according to the *We* by adjusting the *We* when the water droplets collide with a superhydrophobic surface of a nanostructure [43]. In this case, on the superhydrophobic surface, the water droplets showed rebound behavior, and when the *We* increased to 200 or more, the water droplets showed aggregation behavior. In this study, we attempted to compare the *We* on the surface before and after treatment to confirm the change in the behavior of droplets according to the change in each *We*. For comparison, we calculated the *We* during the specimen–water drop collision by using *We* = *ρv*_0_^2^*D*_0_/*γ*. Table 2 shows the *We* before and after treatment. The results of the high-speed camera during the sample-water drop collision are shown in Fig 4(b).

**Table 2:** Interface tension and *We* of the surface before and after treatment.

Treated method	Droplet velocity, *v*_0_	Droplet size, *D*_0_	Density (25℃), *ρ*	Surface energy, *γ*_*sv*_	Surface tension, *γ*_*lv*_	We number, *We*
	[m/s]	[mm]	[Kg/m^3^]	[mN/m]	[mN/m]	[–]
Pristine	1.02	2.8±0.2	998.2	16.39	45.70	63.2
PE				0.76	68.17	42.4
RIE				0.02	73.88	39.1

As shown in Fig 4(c), small air bubbles were generated in the water droplets after the water droplets bounced off the PE specimen. The droplets were transformed into the following shapes and bounced back. In this case, the surface and the droplet had a contact time of 23.5±0.5 ms. The contact time *t*_*c*_ with the surface is calculated as $3.71\sqrt{{\rho r_{0}}^{3}/\gamma }$, and the previous research results were followed [44]. At this time, small droplets were dispersed and dropped from the basic droplets. The diameter of this dispersed droplet was 0.4 mm to 5 mm. The RIE-treated specimen had a shorter contact time of 17.5±0.5 ms ($t_{c}=2.87\sqrt{{\rho r_{0}}^{3}/\gamma }$), although the shape of the droplet at the time of the surface collision was similar to that of the PE-treated specimen. The droplets bounced to a much higher position than the PE treatment and collided with the surface several times. Water droplets dropped from most specimens were removed from the surface by moving the position of the droplets under splashing conditions, and non-removed water droplets were removed from the surface by a slight angle change of 1° or less.

### Self-cleaning effect on the surface treated with PE and RIE methods

**Fig 5:**
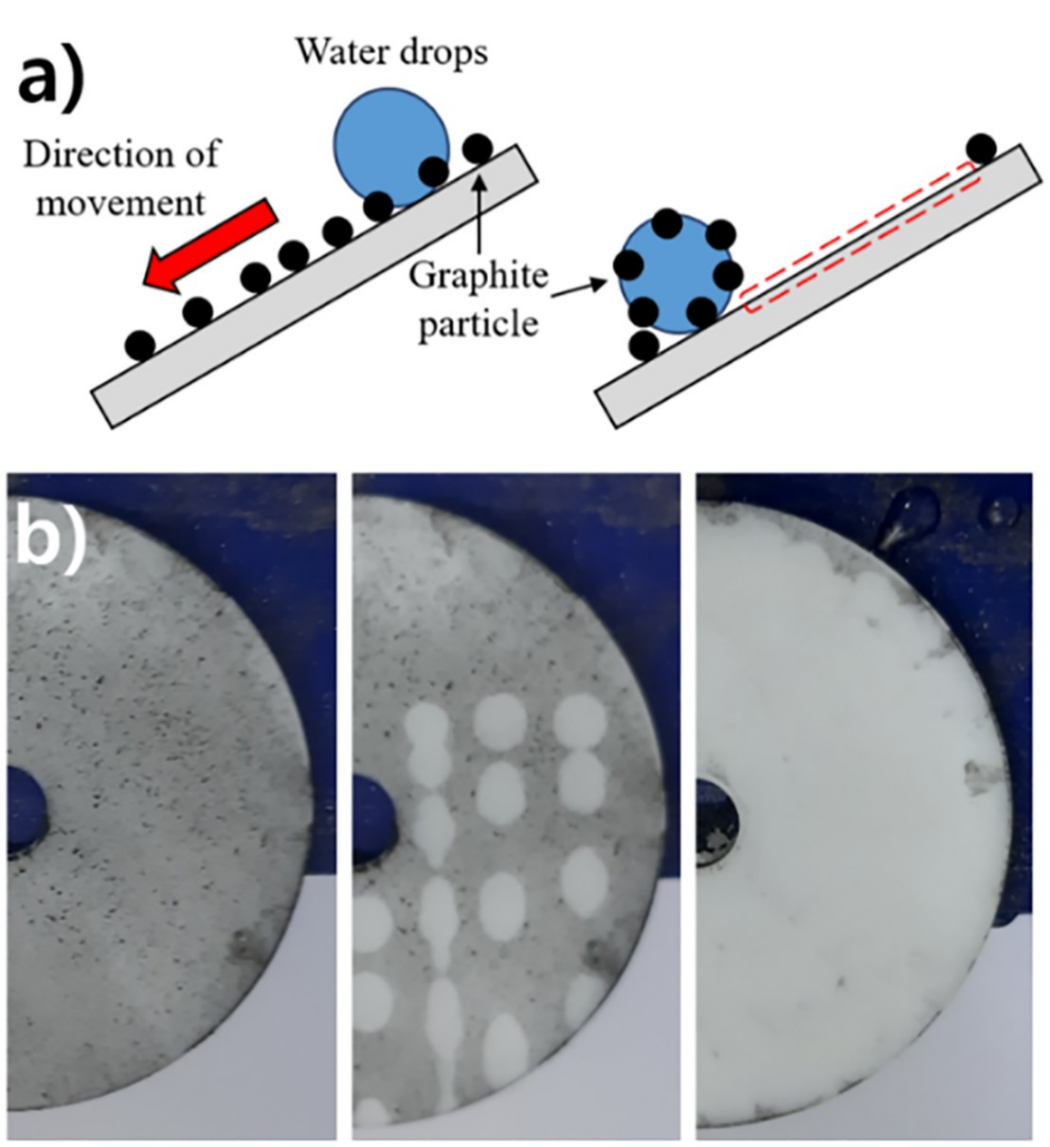
Verifying the self-cleaning effect of the produced specimen. (a) The process of removing fine contaminants from the manufactured specimen. (b) The process of removing contaminants from the treated specimen. The water droplets repeatedly bounced off the surface. In this process, particles in the path were removed, and finally, they were off the surface of the specimen. As a result of repeating the same process for 20 seconds, almost all particles on the surface were removed, and only a few particles remained in the place where water droplets did not contact.

The water repellency of the prepared specimen can help remove contaminants. To confirm this property, the self-cleaning ability of the surface was tested using graphite [45, 46]. After thinly applying graphite (≥45 μm) to the RIE-treated specimen, water droplets using de-ionized water were dropped to remove graphite particles from the surface to clean the surface. As shown in Fig 5(a), the specimen to which the particles were applied was inclined to have a slope of 10°, and the water droplets dropped on the specimen were maintained at a size of 10±0.5 μL.

On the one hand, when water droplets were dropped on the pristine specimens, graphite particles remained on the surface like water droplets. The water droplets absorbed the graphite particles on the surface, but were not removed from the surface. Thus, the particles became dirtier than the initial appearance. When the additional droplets continued to drop, the droplets gathered and were eventually removed from the surface. However, in this process, some liquid remained on the surface and could not be completely removed, and a partially dirty surface could be identified before the drop of water.

On the other hand, water droplets dropped from the specimen after the RIE treatment were removed from the surface while containing graphite particles on the surface. Droplets dropped above 10 mm in height were removed from the specimen by repeatedly bouncing from the surface (Fig 5(b)). The experiment confirmed that when water droplets were continuously dropped, graphite particles were stably removed without leaving water droplets on the surface. As a result, it took only about 20 seconds for the graphite particles to be completely removed from the surface.

## Conclusions

In this study, SHSs were manufactured through PE and RIE methods using O_2_ and Ar plasma, and the characteristics of the two surfaces were confirmed. The experiments confirmed that the PE specimen had water repellency of 150° or more and had a leaf vein-like shape on the surface. When water droplets fell on the surface and bounced back, small water droplets were dispersed. The RIE specimen had a small spherical shape on the surface and had a high contact angle and a low sliding angle. For this reason, it had an excellent self-cleaning effect and water repellency.

## Supporting information

S1 File (ZIP)
