# Supplementary figures and images for: Wettability of polytetrafluoroethylene surfaces by plasma etching modifications
Source: PLoS One. 2023 Mar 31;18(3):e0282352. doi: 10.1371/journal.pone.0282352 (PMC10065265; doi:10.1371/journal.pone.0282352)

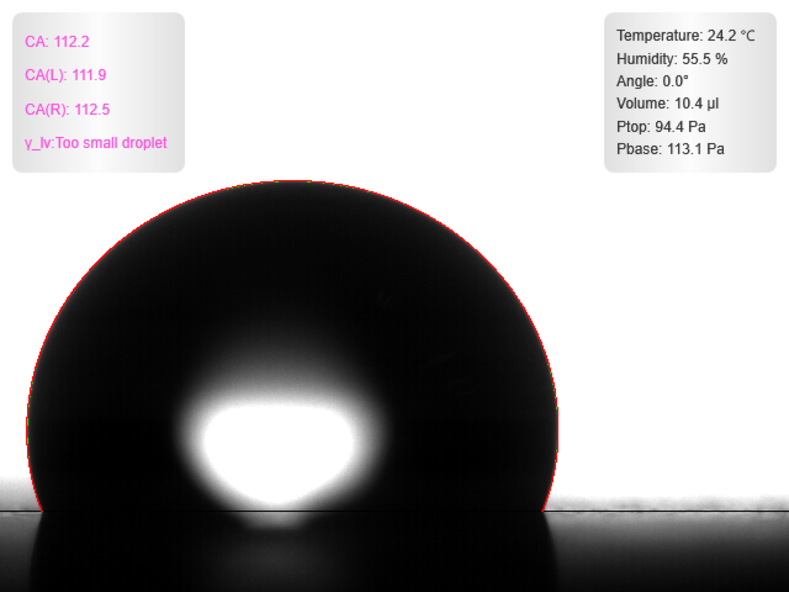

Supplement: S1 File — (ZIP) [file pone.0282352.s001.zip › Fig1_1.tif]

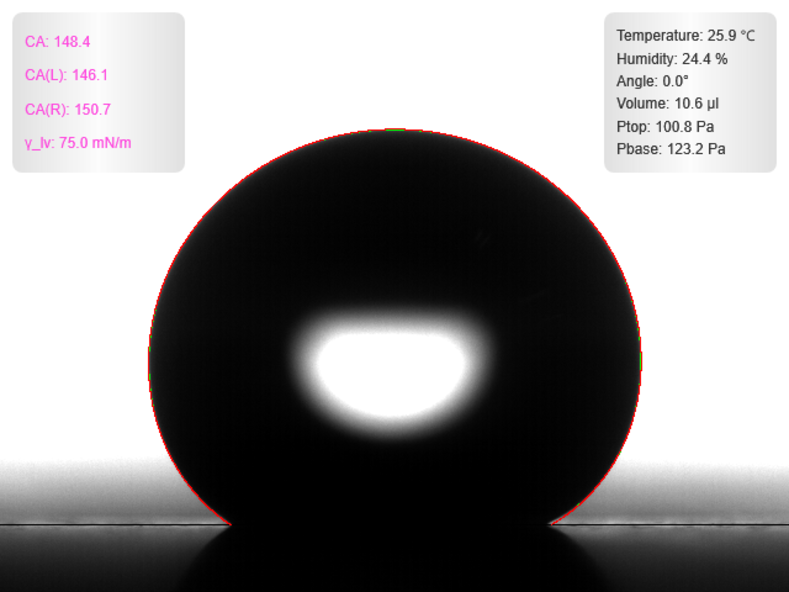

Supplement: S1 File — (ZIP) [file pone.0282352.s001.zip › Fig1_2.tif]

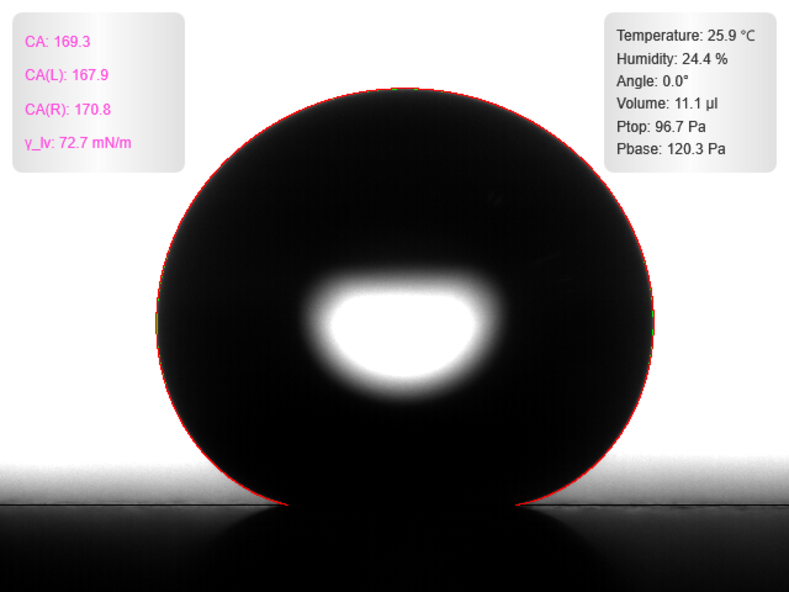

Supplement: S1 File — (ZIP) [file pone.0282352.s001.zip › Fig1_3.tif]

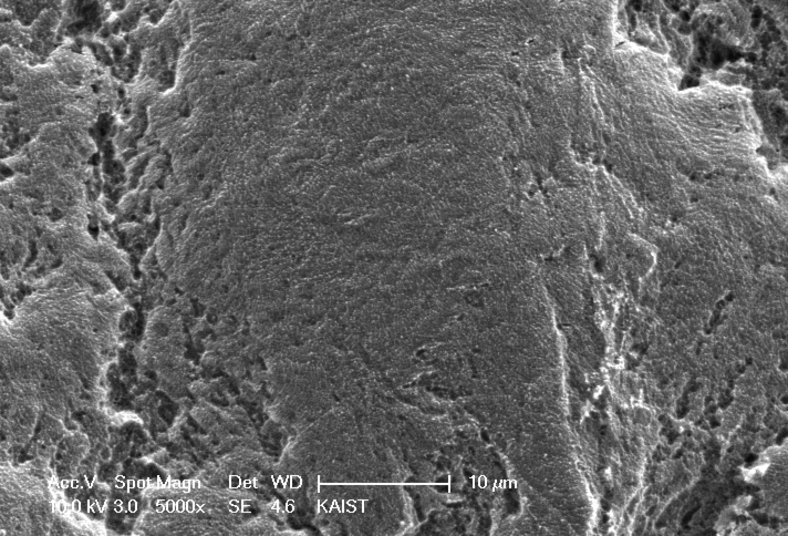

Supplement: S1 File — (ZIP) [file pone.0282352.s001.zip › Fig2_1.tif]

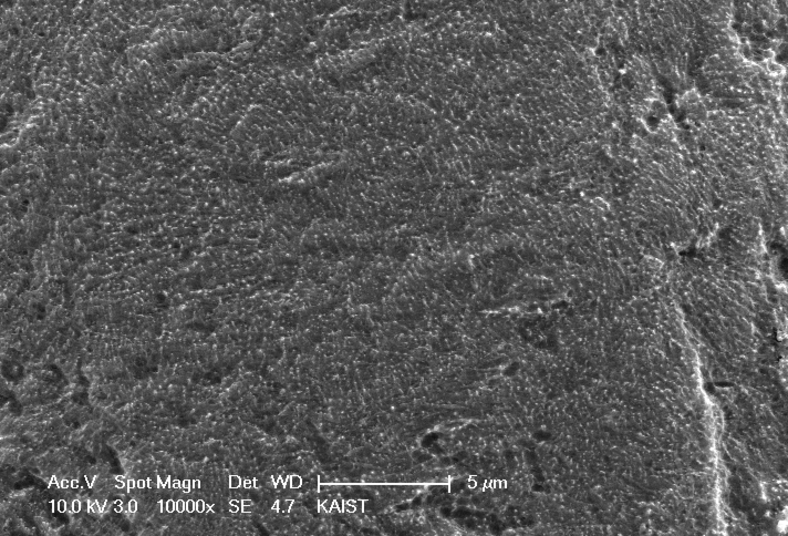

Supplement: S1 File — (ZIP) [file pone.0282352.s001.zip › Fig2_2.tif]

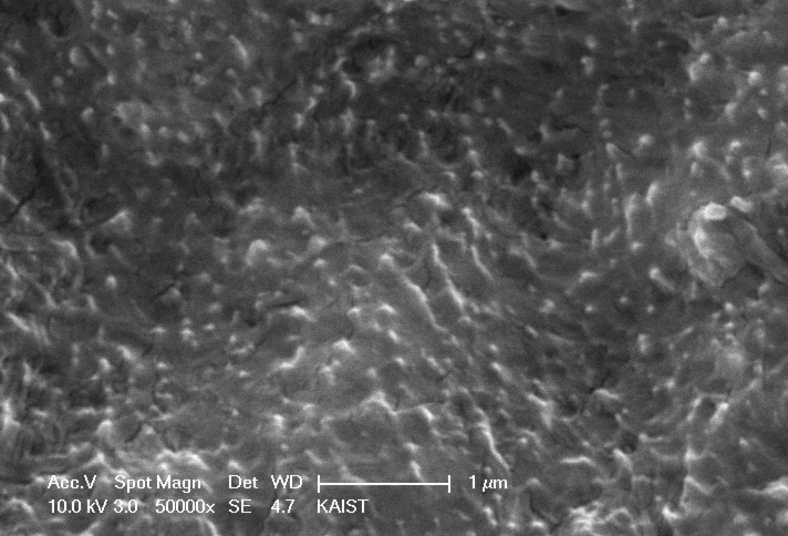

Supplement: S1 File — (ZIP) [file pone.0282352.s001.zip › Fig2_3.tif]

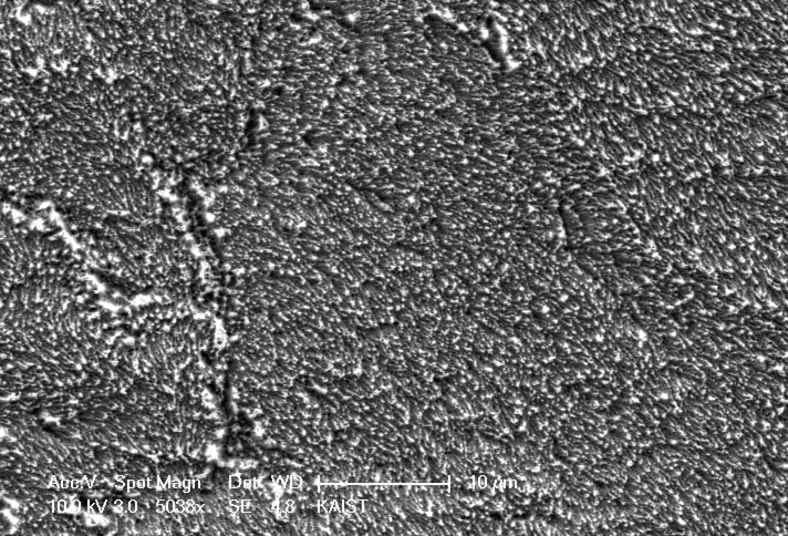

Supplement: S1 File — (ZIP) [file pone.0282352.s001.zip › Fig2_4.tif]

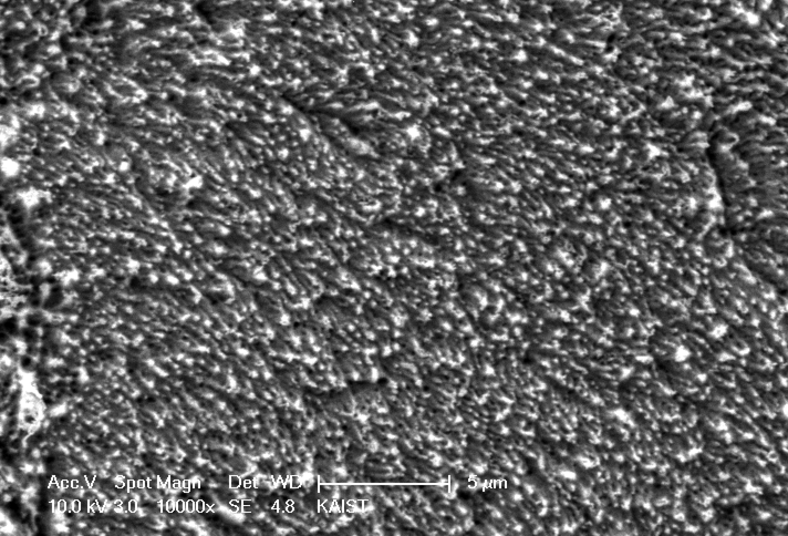

Supplement: S1 File — (ZIP) [file pone.0282352.s001.zip › Fig2_5.tif]

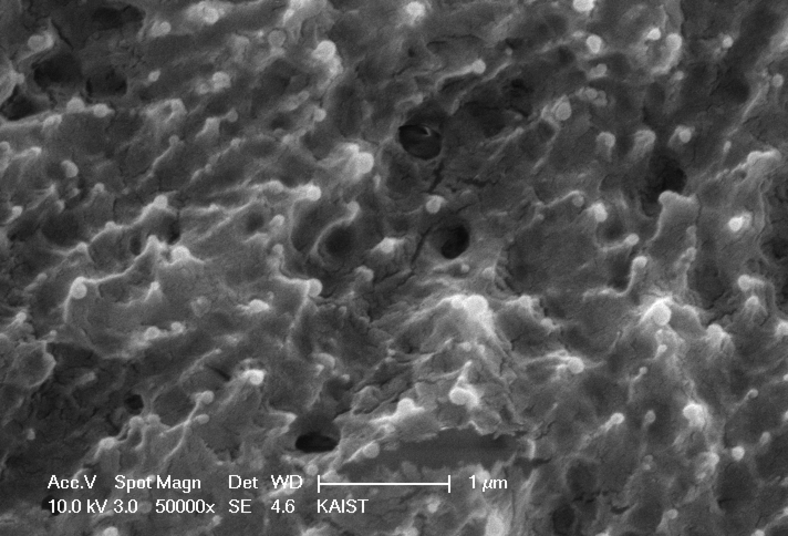

Supplement: S1 File — (ZIP) [file pone.0282352.s001.zip › Fig2_6.tif]

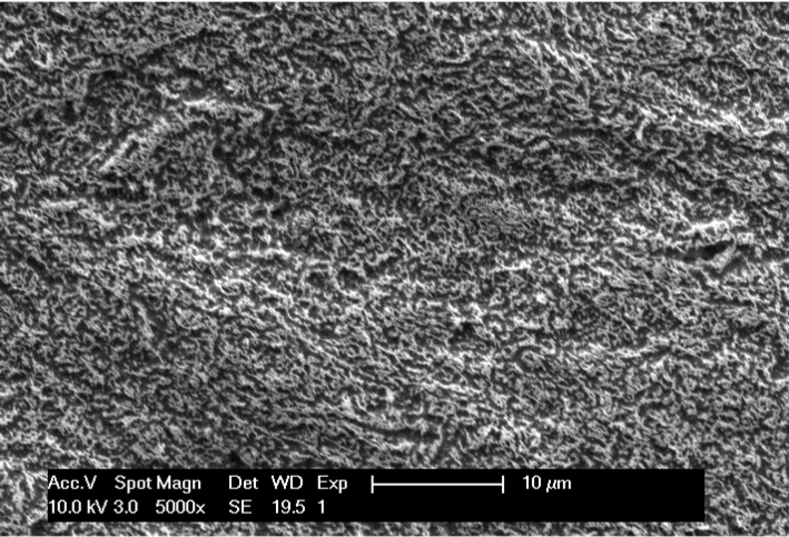

Supplement: S1 File — (ZIP) [file pone.0282352.s001.zip › Fig2_7.tif]

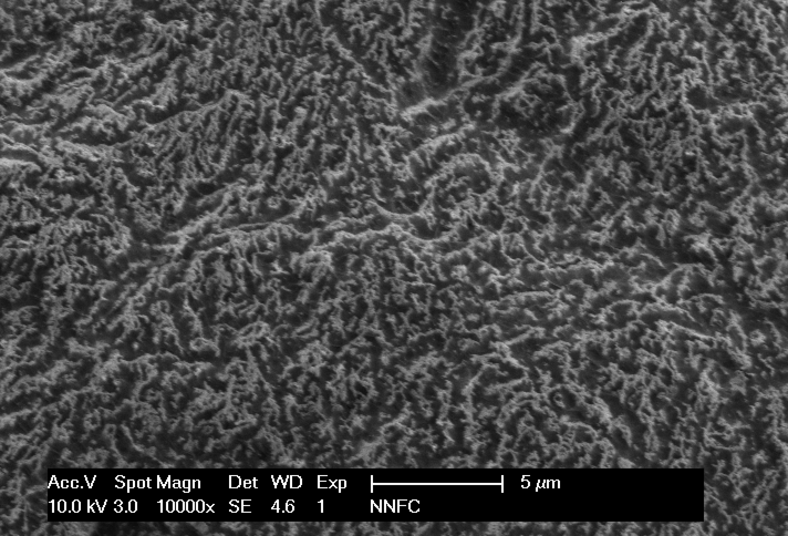

Supplement: S1 File — (ZIP) [file pone.0282352.s001.zip › Fig2_8.tif]

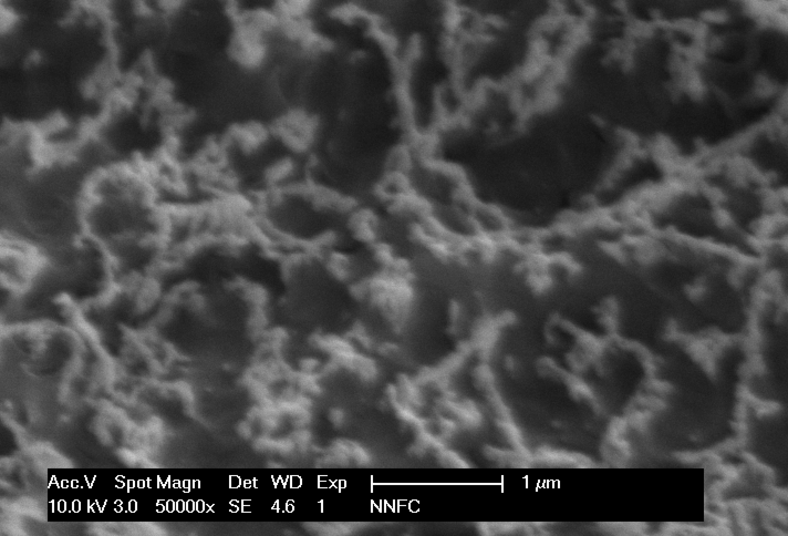

Supplement: S1 File — (ZIP) [file pone.0282352.s001.zip › Fig2_9.tif]

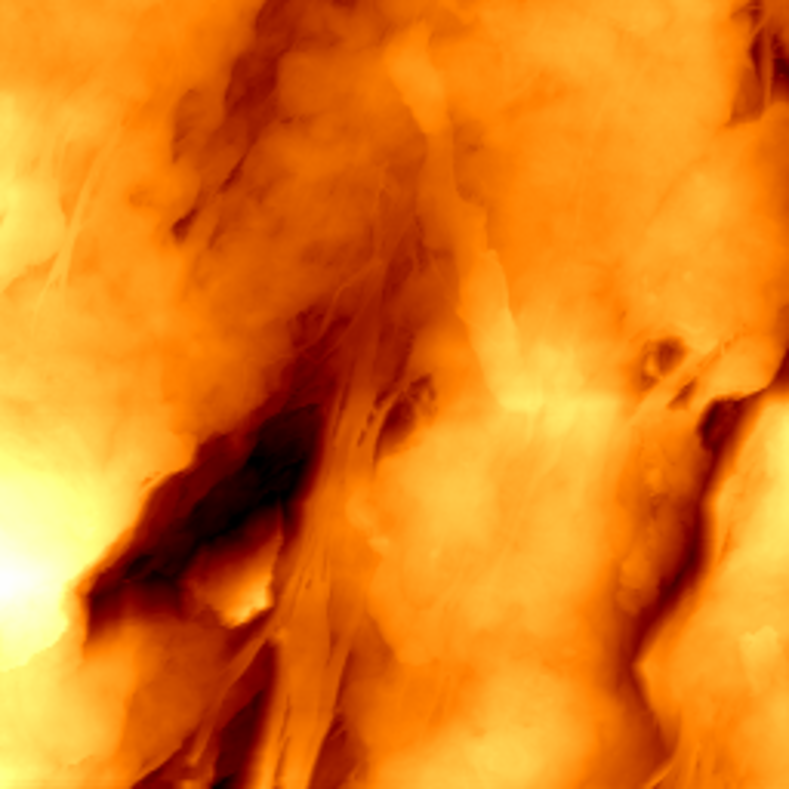

Supplement: S1 File — (ZIP) [file pone.0282352.s001.zip › Fig3_1.tif]

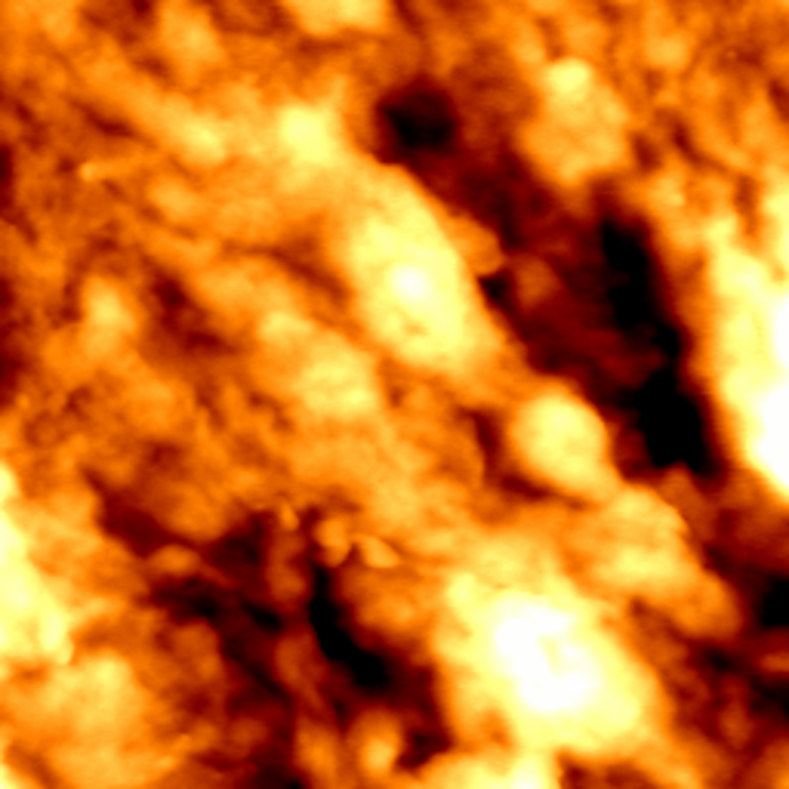

Supplement: S1 File — (ZIP) [file pone.0282352.s001.zip › Fig3_2.tif]

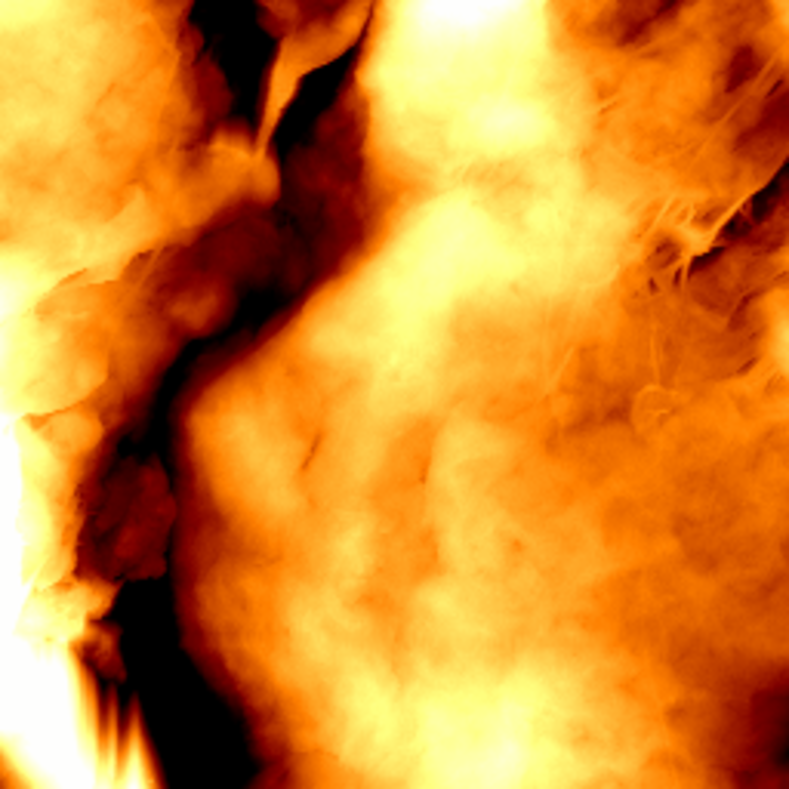

Supplement: S1 File — (ZIP) [file pone.0282352.s001.zip › Fig3_3.tif]

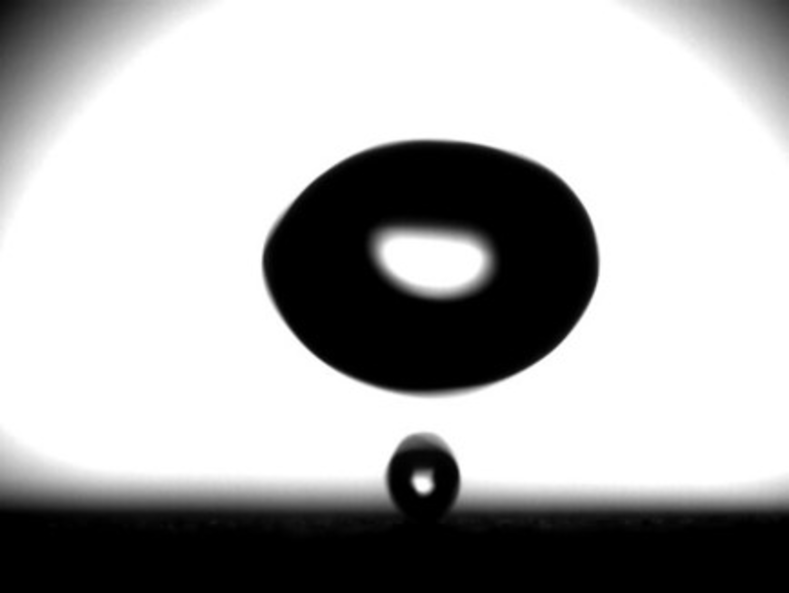

Supplement: S1 File — (ZIP) [file pone.0282352.s001.zip › Fig4_1.tif]

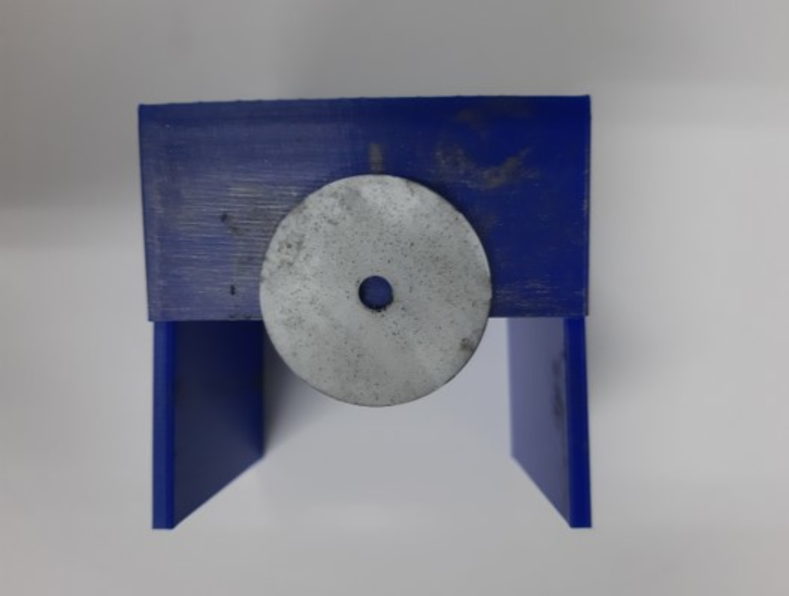

Supplement: S1 File — (ZIP) [file pone.0282352.s001.zip › Fig5_1.tif]

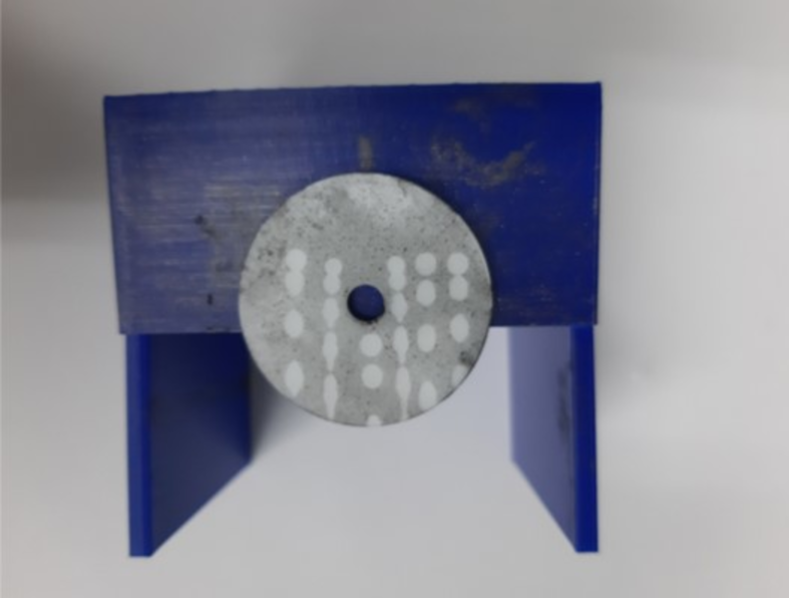

Supplement: S1 File — (ZIP) [file pone.0282352.s001.zip › Fig5_2.tif]

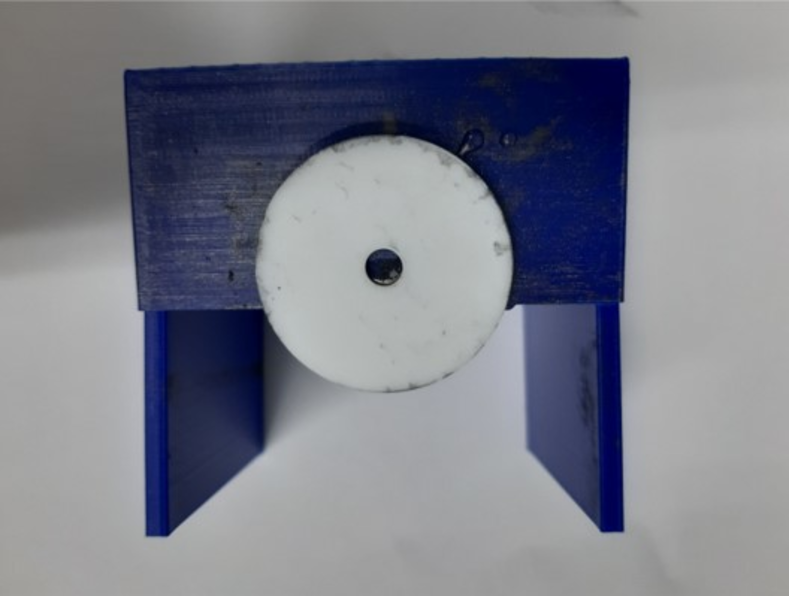

Supplement: S1 File — (ZIP) [file pone.0282352.s001.zip › Fig5_3.tif]
